# Peak Pair Pruner: a post-processing software to MS-DIAL for peak pair validation and ratio quantification of isotopic labeling LC-MS(/MS) data

**DOI:** 10.1093/bioadv/vbad044

**Published:** 2023-03-27

**Authors:** Ryan A Smith, Qibin Zhang

**Affiliations:** Department of Chemistry & Biochemistry, University of North Carolina at Greensboro, Greensboro, NC 27402, USA; Center for Translational Biomedical Research, University of North Carolina at Greensboro, North Carolina Research Campus, Kannapolis, NC 28081, USA; Department of Chemistry & Biochemistry, University of North Carolina at Greensboro, Greensboro, NC 27402, USA; Center for Translational Biomedical Research, University of North Carolina at Greensboro, North Carolina Research Campus, Kannapolis, NC 28081, USA

## Abstract

**Motivation:**

Isotopic labeling is an essential relative quantification strategy in mass spectrometry-based metabolomics, ideal for studying large cohorts by minimizing common sources of variations in quantitation. MS-DIAL is a free and popular general metabolomics platform that has isotopic labeling data processing capabilities but lacks features provided by other software specialized for isotopic labeling data analysis, such as isotopic pair validation and tabular light-to-heavy peak ratio reporting.

**Results:**

We developed Peak Pair Pruner (PPP), a standalone Python program for post-processing of MS-DIAL alignment matrixes. PPP provides these missing features and innovation including isotopic overlap subtraction based on a light-tagged pool sample as quality control. The MS-DIAL+PPP workflow for isotopic labeling-based metabolomics data processing was validated using light and heavy dansylated amino acid standard mixture and metabolite extract from human plasma.

**Availability and implementation:**

Peak Pair Pruner is freely available on Github: https://github.com/QibinZhangLab/Peak_Pair_Pruner. Raw MS data and .ibf files analyzed are on Metabolomics Workbench with Study ID ST002427.

**Contact:**

q_zhang2@uncg.edu

**Supplementary information:**

Supplementary data are available at *Bioinformatics Advances* online.

## 1 Introduction

An essential approach for metabolomics studies is stable isotopic labeling, in which analytes in one sample are derivatized with a ‘light’ tag and those in another sample with a ‘heavy’ or isotopically enriched tag. These samples undergo pooling and mixing strategies to allow for relative quantification in large cohort studies. MS-DIAL (Tsugawa *et al.*, 2015) is one of the most popular general metabolomics analysis platforms (Rakusanova, 2023) and is seeing increasing usage, referenced in 386 articles in 2020, 713 in 2021 and 970 in 2022, respectively on Google Scholar (searched on January 6, 2023). It is free and versatile, even annotating metabolites with user-generated tandem mass spectrometry libraries, but its features in handling isotopic labeling data fall short of specialized software. IsoMS (Zhou *et al.*, 2014) is one such specialized software, designed for the dansylation chemistry-based derivatization (Guo and Li, 2009). IsoMS performs pairing of isotopic peaks and identification based on accurate mass and retention time, providing a tabular report of metabolites with their light-to-heavy (L/H) ratios in the samples. While IsoMS is robust and well suited for its target chemistry, it is a commercial product and there is a desire for more generalized software analyzing isotopic labeling data. MS-IDF (Wang *et al.*, 2022) presents a non-commercial alternative for isotopic labeling analysis but compares just two chromatograms at a time and so is not suited for large cohort studies with its current release. Leveraging the powerful raw data processing capabilities of MS-DIAL in metabolomics, we built Peak Pair Pruner (PPP) for MS-DIAL post processing, providing the missing, specialized isotopic labeling features with innovation not represented in other software options.

## 2 Methods

**Alignment matrix import.** PPP requires an MS-DIAL alignment matrix. Utilizing keywords and isotopic labeling naming convention, PPP collates metabolite, blank, sample, light QC, heavy QC, mix QC, and replicate data into an internal data array.

**Isotopic screening.** Matching isotopic relationships from MS-DIAL and experimental parameters provided by the user, PPP searches the internal data array for peak pairs that are potentially due to the user’s isotopic labeling experiment, accounting for different charge states and adduct species.

**Peak pair mass validation.** Mass defect filtering is optionally applied based on user-defined upper and lower mass defect limits. Accurate mass difference between paired peaks is validated against user-defined heavy tag shift and mass ppm tolerance.

**Peak pair quantitative corrections.** Background peak values are subtracted utilizing a blank. Isotopic overlap between light and heavy tagged analytes is subtracted utilizing the light pool QC.

**Peak pair QC ratio validation.** Peak pairs are validated against minimum light QC L/H ratio, minimum heavy QC H/L ratio, theoretical mix QC L/H ratio and mix QC L/H ratio tolerance.

PPP was implemented in Python utilizing the PySimpleGUI and XlsxWriter packages. We have exported PPP to a single executable program that is independent of its original Python IDE. Technical details are described in the Supplementary Material and the GitHub ReadMe file.

## 3 Results

**Fig. 1.**
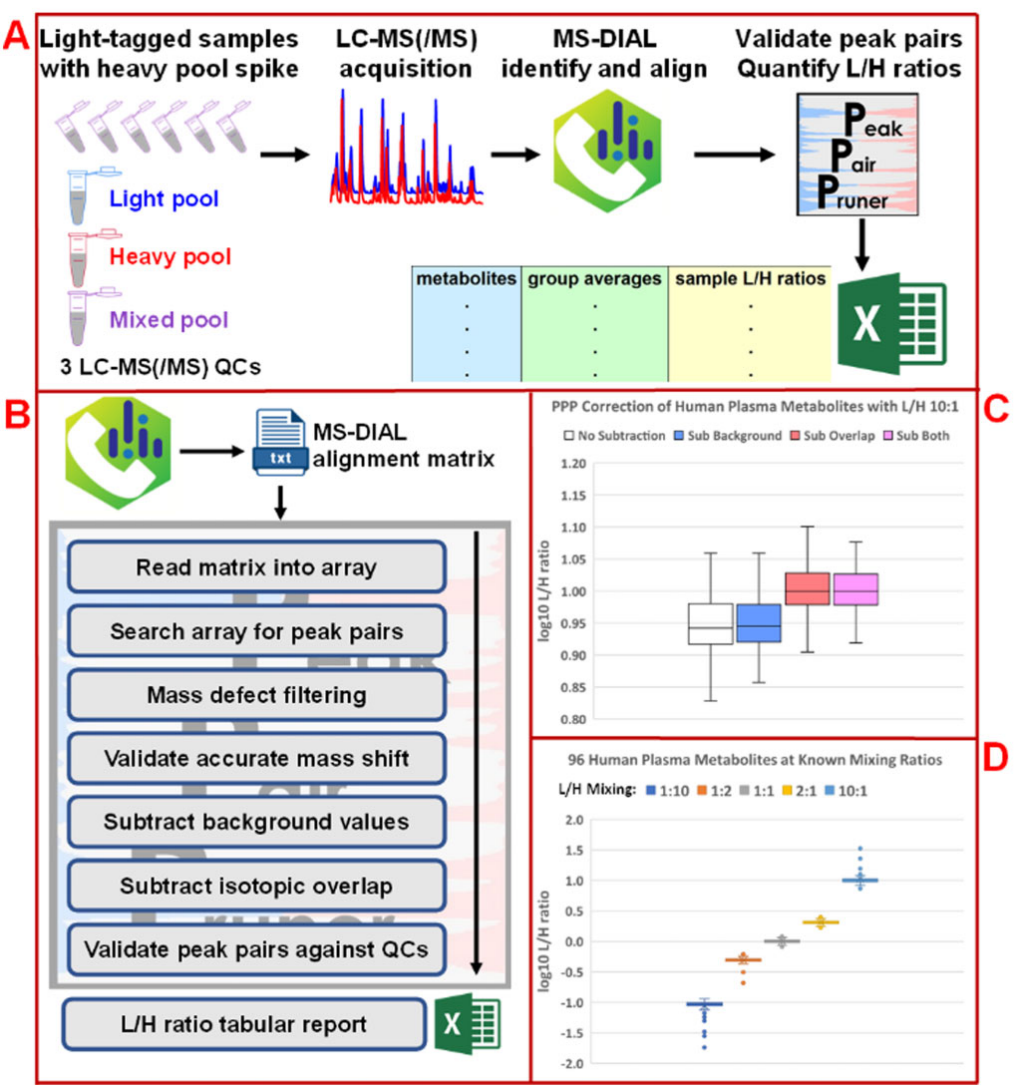
(**A**) MS-DIAL+PPP isotopic labeling workflow in LC-MS metabolomics. Samples are split, mixed to form two pools and aliquots for analysis. One pool is light tagged (light pool QC), while the other is heavy tagged (heavy pool QC). Pools are combined at a known ratio (optimally 1:1) to form the mix pool QC. Analysis aliquots are light tagged and then spiked with heavy pool at the known ratio to make analysis samples. Analysis samples undergo LC-MS(/MS) acquisition, then MS-DIAL peak identification and alignment. PPP performs peak pair validation and quantitative correction based on ratios in the QCs. (**B**) Python coding architecture and processing by PPP. (**C**) L/H 10:1 dansylated human plasma analysis outcomes with and without PPP quantitative corrections. Metabolite peaks may overlap with background peaks, and chemical tags used in light/heavy analysis may overlap in their isotopic envelopes. PPP can optionally correct for background peaks and for isotopic overlap. In the 10:1 L/H sample, the theoretical log10(L/H) value is 1.00, most closely attained when using both corrections. (**D**) Workflow quantitative testing of dansylated human plasma across 100-fold range of theoretical L/H values

To assess quantitation and demonstrate the MS-DIAL+PPP workflow’s capabilities, we conducted two analyses: (i) dansylation of a 17 amino acid standard mixture and (ii) dansylation of pooled human plasma, both with known L/H ratios of 1:10, 1:2, 1:1, 2:1 and 10:1 with high mass resolution LC-MS data acquisition. In analysis (i), all 17 amino acids were identified by MS-DIAL and validated, quantified by PPP. Further details are in the Supplementary Material. In analysis (ii), MS-DIAL produced an alignment matrix with 3501 peaks, among which PPP found 98 identified potential peak pairs and 701 unknown potential peak pairs. PPP validated 96 identified peak pairs and 378 unknown peak pairs. The drop in unknown peak pairs shows the capability of PPP in validation and removing false positive peak pair identifications, a common issue in metabolomics-related database search. Figure 1 illustrates (Figure 1A) the total isotopic labeling workflow with MS-DIAL+PPP, (Figure 1B) PPP coding architecture, and (Figure 1C and D) the key results of analysis (ii). Figure 1C illustrates the usefulness of isotopic overlap subtraction where in the L/H 10:1 sample, the peak pair ratio log10 values are much closer to the expected value of 1.00 following correction. Figure 1D illustrates the accuracy of MS-DIAL+PPP quantitation over a range of metabolite L/H ratios. Noteworthy is the tailing of values at the extreme ratios of 1:10 and 10:1, indicating that these ratios are close to the limit of quantitation for low-abundance metabolites. Further details are in the Supplementary Material.

## Supplementary Material

vbad044_Supplementary_Data
