# Supplementary material for: Peak Pair Pruner: a post-processing software to MS-DIAL for peak pair validation and ratio quantification of isotopic labeling LC-MS(/MS) data
Source: Bioinform Adv. 2023 Mar 27;3(1):vbad044. doi: 10.1093/bioadv/vbad044 (PMC10074028; doi:10.1093/bioadv/vbad044)
Supplement: vbad044_Supplementary_Data [file vbad044_supplementary_data.pdf]

Supplementary Material

Peak Pair Pruner: a post processing software to MS-DIAL for peak pair validation and ratio quantification of isotopic labeling LC-MS(/MS) data

Ryan A. Smith[1,2], Qibin Zhang*[1,2]

1 Department of Chemistry & Biochemistry, University of North Carolina at Greensboro, Greensboro, NC 27402, USA
2 Center for Translational Biomedical Research, University of North Carolina at Greensboro, North Carolina Research Campus, Kannapolis, NC 28081, USA

*Correspondence: q_zhang2@uncg.edu

Materials and Methods

Chemicals and materials. Pooled K2EDTA human plasma (LOT#: HMN654203) was purchased from BioIVT. Amino acid standard mixture (product AAS18-10ML) and sodium carbonate were purchased from Sigma-Aldrich. MS pure $^{13}C_2$-dansyl chloride and $^{12}C_2$-dansyl chloride were purchased from Nova Medical Testing (Edmonton, Alberta, Canada). Other LC/MS grade solvents and reagents were purchased from Thermo Fisher Scientific.

Metabolite sample preparation. The dansyl chloride isotopic labeling protocol(Guo and Li, 2009) was adapted as follows to label human plasma and a 17 amino acid standard mixture. Briefly, metabolites were extracted from a pooled BioIVT human plasma sample using methanol precipitation. The extract was dried and reconstituted in 50% acetonitrile for isotopic labeling. Amino acid standard mixture was also dried first before reconstitution in 50% acetonitrile. Aliquots were then labeled with either the light $^{12}C_2$-dansyl chloride or the heavy$^{13}C_2$-dansyl chloride. After quenching the reaction mixture with 250 mM NaOH, light and heavy labeled samples were recombined to create pools with 1:10, 1:2, 1:1, 2:1, and 10:1 mixing ratios to assess quantitation accuracy.

Mass spectrometry analysis. A Thermo Scientific Vanquish Horizon UHPLC System coupled with a Thermo Scientific Q Exactive HF Orbitrap was used to conduct LC-(+)FTMS analysis. A Waters ACQUITY UPLC BEH C18 column (1.7 µm, 2.1 x 100 mm) was used along with the following mobile phases: (A) 0.1% formic acid in water and (B) 0.1% formic acid in acetonitrile. The gradient was 25% B (0 min), 25% B (1.2 min), 99% B (11.2 min), 99% B (16.2 min), 25% B (16.3 min), and 25% B (19 min) to achieve column equilibration. The column was maintained at 40 ºC with flowrate of 400 µL/min. The injection volume was 5.00 µL. Each mixture and QC was sampled in triplicate. Ionization for FTMS analysis utilized a HESI source in the positive mode with spray voltage 3 kV, capillary temperature 300 ºC, sheath gas flowrate 35 au, aux gas flowrate 10 au, probe heater temperature 300 ºC, and S-lens RF level 50 V. FTMS scanning was full MS1 in the range 250 to 1000 m/z with maximum IT 200 ms, AGC target $5x10^5$, and mass resolution 60,000.

MS-DIAL peak pair identification. MS-DIAL 4.92 for Windows x64 was used alongside an in-house metabolite dansylation library. For the amino acid mixture, MS-DIAL data collection proceeded with MS1 tolerance 0.005 Da and maximum charge number 2. The minimum peak height was set to 10^6 with mass slice width 0.05 Da. The peak smoothing method was linear weighted moving average with a smoothing level of 3 scans and a minimum peak width of 5 scans. The Sigma window value was set to 0.1 without exclusion after precursor ions and with isotopic ions w/o MS2Dec preserved. Automated identification was performed with a text file library with retention time tolerance 0.1 min, accurate mass tolerance 0.01 Da, identification score cut off 85%, and only reporting the top hit. Peak alignment was performed in reference to the light pool QC, with retention time tolerance 0.04 min, MS1 tolerance 0.004 Da, removing features based on blank information, sample max / blank average minimum fold change 5, keeping all metabolite features, and gap filling by compulsion. Isotope tracking was performed with ^{13}C as the labeled element, the light pool QC as the non-labeled reference file, and the heavy pool QC as the fully-labeled reference file. After MS-DIAL processing, the alignment results were exported in mgf format, choosing export option ‘raw data matrixes (Height)’, with filtering by blank ion abundances, and filtering by the result of isotope labeled tracking in reference to the heavy pool QC. For the human

plasma samples, the parameters were nearly the same with identification retention time tolerance 0.5 min and alignment MS1 tolerance 0.003 Da. Alignment tolerances needed tightening for the human plasma experiment due to greater matrix complexity.

PPP peak pair validation and ratio quantification. We have compiled PPP into an individual executable program that runs independently of MS-DIAL. The researcher first provides a matrix alignment file from MS-DIAL, which can have quantitative values in the form of peak areas or of average peak heights. PPP reads the alignment file into an internal array. PPP then searches the internal array for peak pairs that could be originated from the researcher's isotopic labeling experiment. This is where the greatest 'pruning' occurs, as the MS-DIAL alignment file may have tens of thousands of aligned peaks, most of which are unrelated to isotopic labeling. Next, PPP conducts mass defect filtering, keeping peak pairs with mass defects within a window set by a minimum and maximum mass defect. PPP then validates the accurate mass shift between the peaks of each peak pair, checking that the mass difference conforms to the light and heavy labels within a given ppm tolerance. Next comes background subtraction based on the blank, followed by subtraction of heavy isotope overlap based on the light pool QC. Subtraction of the natural heavy abundance is done through the following equations:

$$\text{Equation 1: } R_{natural\ heavy} = \frac{S_{heavy,light\ QC}}{S_{light,light\ QC}}$$

$$\text{Equation 2: } S_{heavy,corrected} = S_{heavy,measured} - S_{light} R_{natural\ heavy}$$

In Equation 1, the natural heavy abundance per light abundance is established from the light pool QC. In Equation 2, the natural heavy abundance for each sample, mix QC, and replicate is subtracted based off the light peak measured in that particular injection. Both subtractions reduce peak pair values to a minimum of zero and are optionally selected by the user such that four choices are possible: no subtraction, background subtraction, overlap subtraction, and both subtractions. If both are selected, background subtraction is applied before overlap subtraction. After quantitative corrections, peak pairs face a final validation against the L/H ratios of the QCs: a minimum L/H ratio in the light pool QC, a minimum H/L ratio in the heavy pool QC, and the L/H ratio in the mix pool QC related to experimental design with a specified tolerance window. Validated peak pairs are outputted as a Microsoft Excel spreadsheet file with metabolite information, group average L/H values, sample L/H values, and mixed pool QC L/H values.

For the amino acid mixture, PPP validated peak pairs in the MS-DIAL alignment result raw data matrix (Height) with the following parameters: mass defect window floor -450 mDa, mass defect window ceiling 300 mDa, minimum L/H ratio in the light pool QC 10.0, minimum L/H ratio in the heavy pool QC 100.0, theoretical mix pool QC L/H ratio 1.0, mix pool QC L/H tolerance 0.2, 1 and 2 tags per molecule, exact peak pair mass shift 2.00671 Da, mass shift tolerance 10.0 ppm, background subtraction enabled, and isotopic overlap subtraction enabled. For the human plasma, the PPP parameters were nearly identical with a mass defect window floor -500 mDa and a mass defect window ceiling 499 mDa. The mass defect window [-500 mDa, 499 mDa] effectively disables mass defect filtering, which is the appropriate choice in the absence of a target metabolite class or *a priori* knowledge of the sample.

Results and Discussion

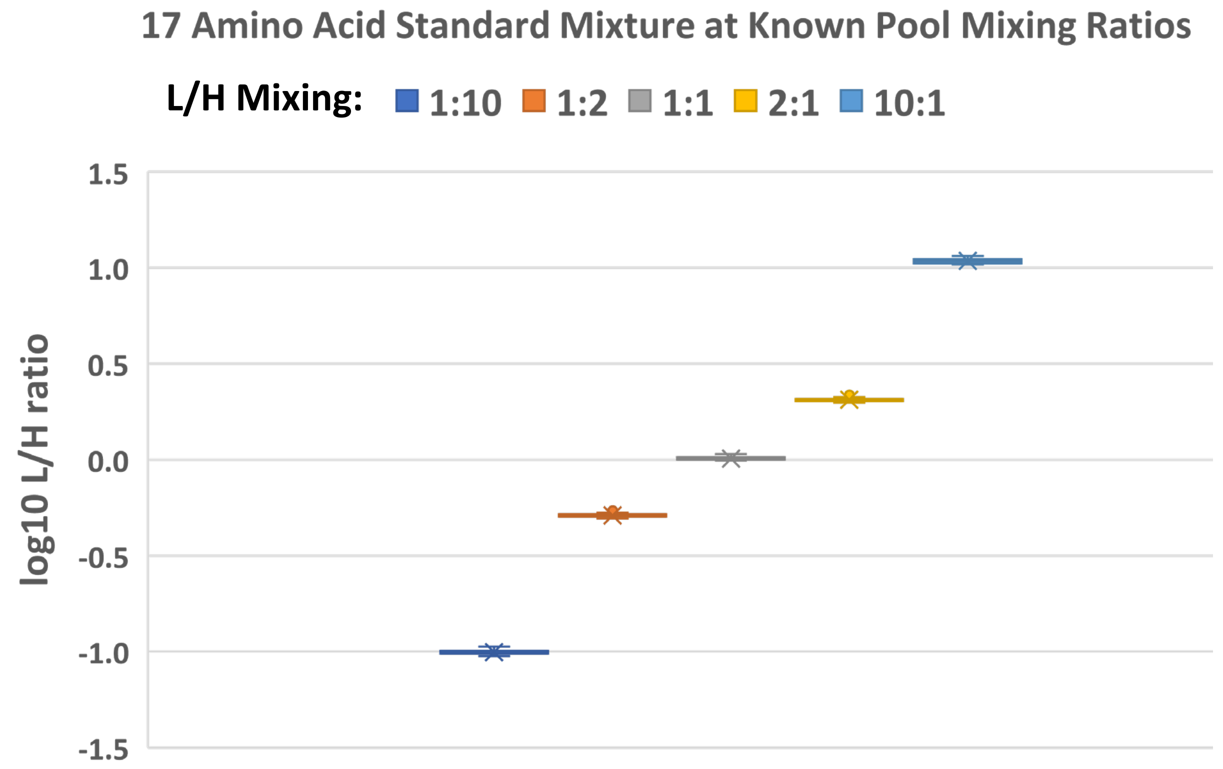

Figure S1: Boxplot representation of MS-DIAL+PPP analysis of dansylated AA standard mixture. Light (L) and heavy (H) pools were mixed at 1:10, 1:2, 1:1, 2:1, and 10:1 to assess workflow quantification with expected $\log_{10}$(L/H) values of -1.0, -0.3, 0.0, 0.3, and 1.0, respectively.

Dansylated amino acids. Figure S1 illustrates the results of the amino acid dansylation experiment. The amino acid standard mixture from Sigma Aldrich contains 17 amino acids. All 17 light-tagged amino acids were automatically identified in MS-DIAL with an the inhouse library. They were paired with M+2 isotopic peaks in the case of singly tagged amino acids and M+4 isotopic peaks in the case of doubly tagged amino acids along with other experiment-unrelated isotopic relationships. PPP then validated all 17 experimental peak pairs and reported the amino acid L/H ratios in each mixture sample. The PPP-reported L/H values conform very nicely to the experimental pool mixing ratios. As the expected L/H ratios cover a 100-fold range in this experiment, these values were transformed to $\log_{10}$ values in Excel for presentation.

References

Guo, K. and Li, L. Differential 12C-/13C-isotope dansylation labeling and fast liquid chromatography/mass spectrometry for absolute and relative quantification of the metabolome. *Anal Chem* 2009;81(10):3919-3932.
